# Scaling up public mental health care in Sub-Saharan Africa: insights from infectious disease

**DOI:** 10.1017/gmh.2021.41

**Published:** 2021-11-11

**Authors:** Susan M. Meffert, Collene Lawhorn, Linnet Ongeri, Elizabeth Bukusi, Holly R. Campbell, Eric Goosby, Stefano M. Bertozzi, Simon Njuguna Kahonge

**Affiliations:** 1 Department of Psychiatry, University of California, San Francisco (UCSF), San Francisco, California, USA; 2 Communication, Dissemination and Engagement Research Program for HIV Prevention, Treatment and Cure, Division of AIDS Research, National Institute of Mental Health (NIMH), Bethesda, Maryland, USA; 3 Kenya Medical Research Institute (KEMRI), Nairobi, Kenya; 4 Departments of Obstetrics and Gynecology, University of Washington, Seattle, Washington, USA; 5 Center for Microbiology Research, KEMRI, Nairobi, Kenya; 6 Department of Obstetrics and Gynecology, Aga Khan University, Nairobi, Kenya; 7 Human Mobility and HIV Research Program Division of AIDS Research, NIMH, Bethesda, Maryland, USA; 8 NIMH Center for Global Mental Health Research, Bethesda, Maryland, USA; 9 Department of Internal Medicine, UCSF, San Francisco, California, USA; 10 United Nations (Special Envoy on Tuberculosis), Washington, DC, USA; 11 School of Public Health, University of California at Berkeley (UCB), Berkeley, California, USA; 12 Division of Mental Health, Ministry of Health (MOH) Republic of Kenya, Nairobi, Kenya

**Keywords:** HIV, mental health, health policy, treatment cascade, Sub-Saharan Africa

## Abstract

**Introduction:**

Models estimate that the disability burden from mental disorders in Sub-Saharan Africa (SSA) will more than double in the next 40 years. Similar to HIV, mental disorders are stigmatized in many SSA settings and addressing them requires community engagement and long-term treatment. Yet, in contrast to HIV, the public mental healthcare cascade has not been sustained, despite robust data on scalable strategies. We draw on findings from our International AIDS Society (IAS) 2020 virtual workshop and make recommendations for next steps in the scale up of the SSA public mental healthcare continuum.

**Discussion:**

Early HIV surveillance and care cascade targets are discussed as important strategies for HIV response in SSA that should be adopted for mental health. Advocacy, including engagement with civil society, and targeted economic arguments to policymakers, are reviewed in the context of HIV success in SSA. Parallel opportunities for mental disorders are identified. Learning from HIV, communication of strategies that advance mental health care needs in SSA must be prioritized for broad global audiences.

**Conclusions:**

The COVID-19 pandemic is setting off a colossal escalation of global mental health care needs, well-publicized across scientific, media, policymaker, and civil society domains. The pandemic highlights disparities in healthcare access and reinvigorates the push for universal coverage. Learning from HIV strategies, we must seize this historical moment to improve the public mental health care cascade in SSA and capitalize on the powerful alliances ready to be forged. As noted by Ambassador Goosby in our AIDS 2020 workshop, ‘The time is now’.

## Introduction

### HIV and mental health in Sub-Saharan Africa: shared goals, divergent paths

HIV plays an important role in the public health of Sub-Saharan Africa (SSA). In recent decades, HIV researchers, health policymakers, and funders collaborated to advance the HIV treatment cascade in SSA, reducing population-level HIV mortality and morbidity. Partially reflecting their success, the burden of human disease in SSA is shifting from communicable to non-communicable disorders, with a substantial contribution from mental disorders (Gouda *et al*., 2019). Epidemiologic models estimate that the disability burden from mental disorders in SSA will increase by 130% over the next 40 years (Charlson *et al*., 2014). Similar to HIV, mental disorders are stigmatized in many SSA settings and addressing the burden of mental disorders requires community engagement and long-term treatment. However, *in stark contrast to HIV*, care and treatment cascades for mental health have not expanded across the SSA public sector care continuum, despite nearly 20 years of epidemiological, efficacy and effectiveness data demonstrating that local non-specialists are capable of successful mental health care delivery for common mental health disorders in SSA (Bolton *et al*., 2003; Lancet Global Mental Health Group *et al*., 2007; van Ginneken *et al*., 2021). Despite progress toward integrated models of mental health treatment delivered within HIV care (Collins *et al*., 2006; Chibanda *et al*., 2015; Chuah *et al*., 2017), the scale up of mental health services that are not linked to HIV care and/or embedded with other services still lags and has much to learn from HIV implementation methods.

Mental health care needs are amplified in the context of coronavirus disease-2019 (COVID-19) – the rising deaths, social gathering restrictions, school closures, economic downturn, and uncertainties of resolution have contributed to increased prevalence of depression and anxiety (Pfefferbaum and North, 2020). There is now attention to mental health care needs across stakeholder groups, including the media, civil society, service-users, healthcare providers, and policymakers (Jones *et al*., 2020; Moreno *et al*., 2020). In July of 2020, as part of the International AIDS Conference (AIDS 2020), we convened a scientific workshop entitled, ‘Why HIV and Mental Health Care Need to Work Together in Sub-Saharan Africa: Collaborative Scale-Up to Address Evolving Epidemics’, which brought together researchers, policy experts, and funders to share insights on the prevalence of mental disorders in HIV-affected populations in SSA; explore opportunities for collaborative mental health-HIV implementation; and identify research needs. In this paper, we discuss the role of these stakeholders for advancing the effort, outline progress in the care continua for HIV and mental disorders, compare and contrast where lessons from HIV are valuable, and make recommendations for next steps.

## Discussion

### Enhance public mental health surveillance

Response to the HIV epidemic in SSA is distinguished by the combination of scientific advances, political advocacy, and funds mobilization that supported the development of a continuum of HIV care, spanning testing, treatment, and viral suppression. In 2015, the Joint United Nations Program on HIV and AIDS (UNAIDS) set 2020 goals (90-90-90): 90% of HIV-positive people know their status, 90% of all persons living with HIV (PLWH) are on anti-retroviral therapy, and 90% of all PLWH are virally suppressed. For countries with the high numbers of new HIV infections, including many in SSA, the 2015 announcement was supplemented by the fast-track, 95-95-95 goals to be attained by 2030 (UNAIDS, 2015).

**Fig. 1.**
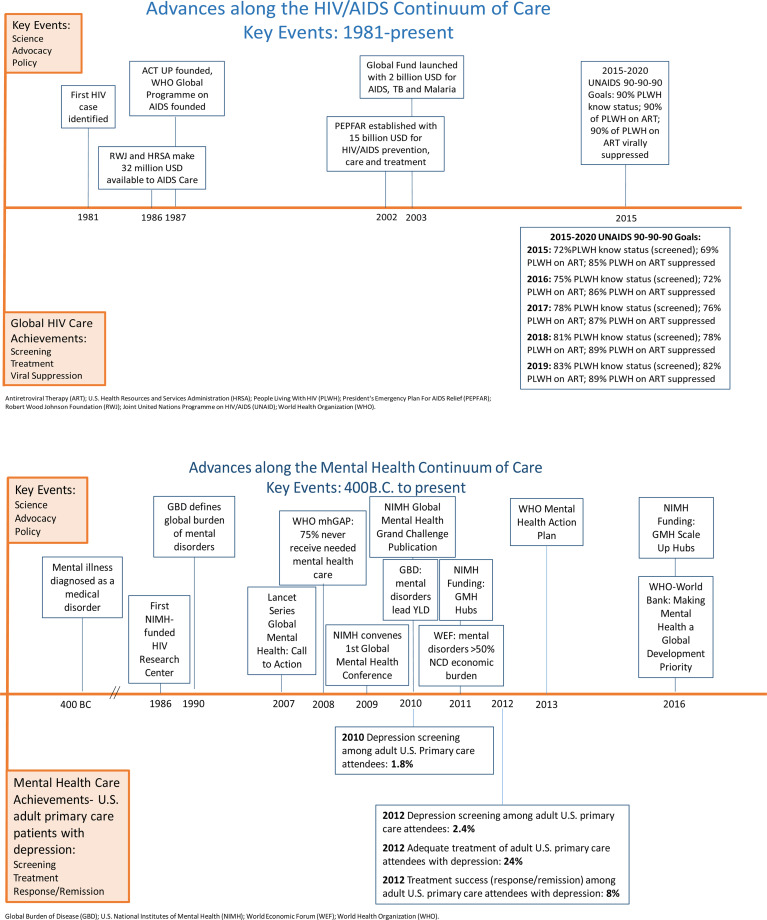
Mental Health and HIV: Advancement of the Continuum of Care.

In contrast, SSA public sector treatment for mental disorders has remained primarily focused on acute care (e.g. hospitalization for psychosis). There is a dearth of public sector outpatient care, even for the most prevalent mental health disorder – depression – which generates the majority of global mental health disability. In low and middle-income countries (LMICs), an extraordinary 76–85% of people with serious mental disorders (including depression) are never treated (World Health Organization, 2013). Mental disorder screening and monitoring of treatment success remain vastly underdeveloped. Below, we compare the timelines of scientific progress, advocacy, political action/policy change, and funding between HIV and mental health. Given the paucity of measurement data for mental health care cascades, we use global adult HIV statistics (UNAIDS) and US adult primary care clinic attendees with depression (Fig. 1) (Pence *et al*., 2012; Akincigil and Matthews, 2017; ‘HIV estimates with uncertainty bounds, 1990–2019’, 2020). We note that this discrepancy should favor results for mental health, given the healthcare resources in high-income countries and the study population of adults engaged in healthcare (*v.* general adult populations which may or may not be linked to healthcare).

Timelines for the development of mental health and HIV care continua are different. The first case recognition of HIV was in 1981, and by 2019, global screening, treatment, and viral suppression were achieved for more than 80% of PLWH (Fig. 1). Mental illness was recognized as a medical problem in 400B.C. and, even for US adults engaged in primary care, depression screening, treatment, and treatment success are still low (Fig. 1). Some may argue that the heterogeneity of mental disorder diagnostic categories, disease courses and treatment responses differentiate it from HIV and even throw into question the relevance of the HIV's care implementation lessons to mental health services. However, as the life expectancy of PLWH increases with improved treatment, HIV/AIDS management has successfully evolved from a predominance of acute care, to its current approach to HIV as a chronic disease. With chronic care for PLWH, have come heterogeneous disease courses, driven, for example, by the growing prevalence of non-HIV co-morbidities.

One potential determinant of timeline differences is the HIV community's effort to set care goals with target dates: e.g. 90-90-90 goals (2020 target) and 95-95-95 ‘fast-track’ goals (2030 target). Country-level measurement and reporting on HIV care cascade goals is central to HIV programming. Aspirational goals have been set for mental health, such as the World Health Organization (WHO) Special Initiative on Mental Health: ‘100 million more people have access to quality and affordable mental health care by 2023’, and the Sustainable Development Goals (SDGs) address non-communicable disease targets: ‘By 2030, reduce by one third premature mortality from non-communicable diseases through prevention and treatment and promote mental health and well-being (SDGs, section 3.4)’. However, global mental health has not yet identified clear, specific, and easily quantifiable goals to define progress on ramping up the continuum of care, nor a system for regular country- or regional-level measurement and accountability. Indeed, data on the mental health continuum of care are so scarce as to make progress toward addressing the treatment gap largely unknown. Setting continuum of care goals does not automatically lead to their achievement. Yet, in the absence of goals and measurement of progress toward them, it is hard to galvanize other critical aspects of an effective public health response, such as action from advocates and policymakers.

### Emerging action on mental health in Kenya

Over the past several years, the Kenyan government has shown leadership in recognizing public mental health care needs, planning for scale up, and allocating budget. In 2018, Senator Sylvia Kasanga drafted an amendment to the 1989 mental health bill. The amendment asserted that each level of the government, from national to county levels, must address accessibility of mental health care services including the availability of treatment. Kenyan counties, which hold health care budgetary power, would be represented at the Kenya Mental Health Board. Senator Kasanga's proposed amendment built on an already strong wave of grassroots and social media activism (e.g. Users and Survivors of Psychiatry in Kenya [USPKenya]) and galvanized the Kenya Ministry of Health (MOH) to address mental health care needs. In 2019, President Uhuru Kenyatta commissioned a mental health task force to assess Kenyan mental health. The task force conducted town hall meetings across the country to solicit public input and presented their recommendations for scaling and funding public mental health care to President Kenyatta in early 2020. The demand for better access to public mental health care in Kenya is now propelled by civil society, service-users, government champions, and policymakers. The COVID epidemic and related escalation of mental health care needs are strengthening Kenya's multi-sectoral drive toward improved mental health care. Indeed, Kenya recently released a robust mental health action plan: 2021–2025. The negative impact of COVID on mental health and the need to continue and expand collaboration with civil society, including mental health service user groups such as USPKenya, is underscored [e.g. participation of user organizations in mental health roles at national and all county levels is targeted at 100% by 2022/2023 (Target 1.5 of Mental Health Leadership and Governance Objectives); Kenya Ministry of Health, 2021]

### Increase advocacy from civil society for attention and funding

Political action groups such as the AIDS Coalition to Unleash Power (ACT UP) were pivotal in the early HIV/AIDS response. ACT UP used direct action, protests, and advocacy to galvanize scientists and policymakers toward HIV treatment needs. Early HIV civil action was supported by community ties that facilitated mobilization – e.g. existing social connections between gay men, healthcare workers and those requiring frequent transfusion. In contrast, people living with mental disorders are not typically linked socially in a way that would foster organized political action. Yet, there is broad global agreement across mental health civil society stakeholders from 53 countries that an ‘effective program for encouraging advocacy’ is critical to achieving ‘national mental health policy or strategy’ (Copeland *et al*., 2014). As recently manifested by movements like Black Lives Matter, preexisting social ties are not necessary to mount national and international demonstrations for social and political change. WHO's World Mental Health Day ‘Big Event’, demonstrated how new media tools can be leveraged for online advocacy with global reach (WHO: World Mental Health Day ‘Big Event’, 2020).

### Deliver economic arguments to policymakers

Health economists contributed to the expansion of HIV care in SSA by illuminating the impact of inaction (Booysen, 2004; McDonald and Roberts, 2006; Lovász and Schipp, 2009; Nkomo, 2010; Interests, 2011). Mental disorders, particularly depression, are well known to negatively impact economic productivity and absenteeism (Chisholm *et al*., 2016). A recent study by the International Alliance of Mental Health Research Funders (IAMHRF) reports that the US$3.7 billion spent annually on global mental health research represents an investment of only US$0.50 per person annually (Woelbert *et al*., 2020). The IAMHRF's analysis of mental health investment characterized systemic inequities: investments are primarily from and are spent in high-income countries; underfunding compared to physical diseases; and a lack of focus on specific mental health disorders, clinical/applied research, and youth mental health. Cost-benefit analyses conducted by WHO demonstrate economic benefits and health returns of 3.5–5.7 dollars for every 1 dollar invested in treatment for depression and anxiety, the two most prevalent mental disorders (Chisholm *et al*., 2016). The global mental health research community must continue to engage policymakers on the economic benefits of mental health.

### Adopt new communication frameworks for global mental health

The ‘Lazarus Effect’, as it was communicated early in the HIV epidemic, pertained to the effect of administering antiretroviral therapy to extremely ill patients with AIDS, allowing many to essentially rise from their deathbeds. The before/after pictures were graphic, compelling images that motivated scientists, advocates, policymakers, and funding (Kim and Farmer, 2006).

In the absence of photos of near-death cachexia and treatment remediation, can mental health care in SSA and globally hope to garner the advocacy, political action, and funding that will be required to address the global epidemic? HIV continues to benefit from focused health communication efforts that target various aspects of the HIV care continuum (Tomori *et al*., 2014; Maloney *et al*., 2020). The global health community must also evolve to communicate the complexities of mental health suffering, recovery, and hope. One way to meet this challenge is through communications that integrate sustainable models for global mental health learning and information dissemination. Sharing the personal narratives and insights of those with lived experiences is an opportunity to change perceptions, build empathy, and reduce the stigma around mental illnesses (Byrne, 2017). Translating evidence-based findings and research advancements in mental health for a variety of stakeholders – including those outside of academia, in health and non-health sectors – will increase the understanding of mental illnesses and promote public and policy engagement (Woolley *et al*., 2016; Martinez-Conde *et al*., 2019). We note that biomedical providers are often not the first consulted for mental disorders and recognize the need to engage groups including traditional healers and religious leaders (Burns and Tomita, 2015). Communication frameworks should leverage media to build mental health advocacy through sharing of key messages while promoting professional standards for communication consistency, consensual information-sharing, reciprocal learning, preferred language for reporting, and respectful transparent community engagement. New technologies like social media can grow diverse networks to raise awareness of the importance of mental health, and new partnerships with traditional media can reach those with limited technology access. Mobile health communication interventions are currently in development that would improve migrants' engagement with HIV care during times of mobility (Clouse *et al*., 2020). Global mental health communication frameworks should thoughtfully integrate intercultural competencies, such as neurodiversity, ethnicity, gender, language proficiency, trust, and context to reach the populations most vulnerable to mental illnesses and to positively influence behavior and policy (Faregh *et al*., 2019). This is especially important for migrant communities who lack stable information resources – e.g. migrants, refugees, or forcibly displaced individuals with ongoing trauma(s).

### Apply HIV care cascade principles to mental healthcare research, targets, and timeline in SSA

How can we translate lessons from the success of the HIV care continuum into specific strategies for the SSA mental healthcare cascade? We draw on our above discussion to provide a template of needed research to drive the expansion of public sector mental health care, and an example of how specific targets and timelines might be implemented and assessed for depressed individuals in public sector primary care by adding a workforce of mental health non-specialists trained to screen and deliver evidence-based mental health care at the clinic (van Ginneken *et al*., 2021) (Table 1). We acknowledge that advancing the mental healthcare cascade among primary care patients (who are already linked to healthcare) may be faster and less complex than with the general population. Yet, the vast numbers of patients with undetected depression in SSA public sector primary healthcare make this a strong starting point both for reaching those in need of care, and for building momentum among key stakeholders for future scale up in other populations. We further acknowledge that this is one possible template among many and hope that it generates many more questions than it answers, stimulating discussion and attention to progress on a sustained public mental healthcare cascade in SSA.

**Table 1.** Template: application of HIV care cascade principles to mental health

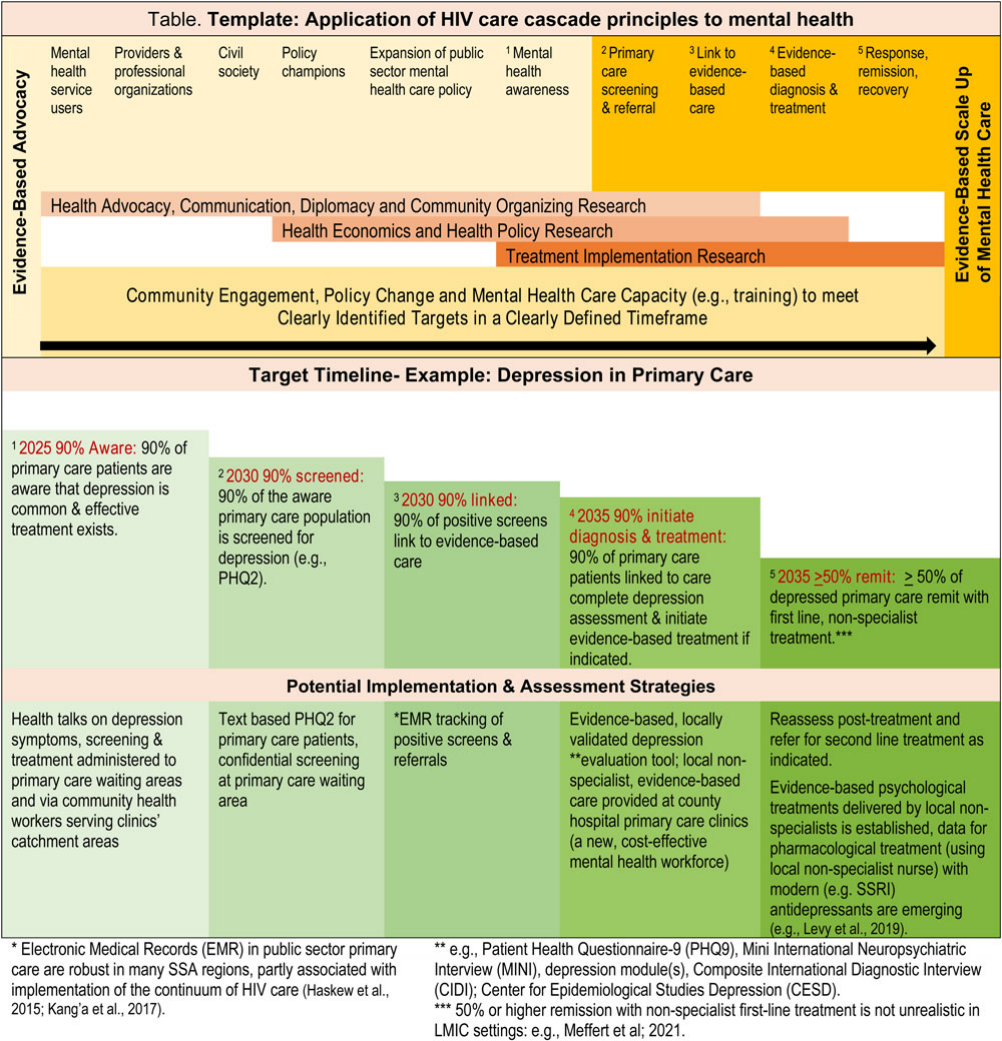

a 50% or higher remission with non-specialist first-line treatment is not unrealistic in LMIC settings: e.g. Meffert *et al*. (2021).

b Electronic Medical Records (EMR) in public sector primary care are robust in many SSA regions, partly associated with the implementation of the continuum of HIV care (Haskew *et al*., 2015; Kang'a *et al*., 2017).

c For example, Patient Health Questionnaire-9 (PHQ9), Composite International Diagnostic Interview (CIDI), Center for Epidemiological Studies Depression (CESD).

## Conclusions

### Recognition of the need for mental health and universal healthcare coverage is acute

The case for universal health coverage has been advanced by COVID-19 (King, 2020). Provided that mental and physical health are granted the parity they deserve, proponents of universal health coverage should serve as valuable partners in the overdue scale up of mental health services. This is a historical moment – mental health care needs are globally recognized across scientific, media, policymaker, and civil society in an unprecedented way and powerful alliances for change are ready to be forged. As noted by Ambassador Goosby in our AIDS 2020 workshop, ‘The time is now’ (Comment from Ambassador Eric Goosby, 2020).
